# Progressive Development of a New Tool for Rapid Thematic Analysis of Community Perceptions and Concerns During Health Emergencies

**DOI:** 10.9745/GHSP-D-24-00281

**Published:** 2025-12-31

**Authors:** Giulia Earle-Richardson, Ciara Nestor, Christine E. Prue

**Affiliations:** a U.S. Centers for Disease Control and Prevention, Atlanta, GA, USA.

## Abstract

**Background:**

Rapid analysis of community needs, perspectives, and concerns during global health emergencies is essential but technically challenging. In the past, emergency responders have struggled to listen to and engage affected communities because of perceptions about anticipated costs and time delays in receiving actionable results.

**Tool Development:**

The U.S. Centers for Disease Control and Prevention Excel Tool for Thematic Analysis was developed over 5 years of assisting with emergency responses for Ebola, COVID-19, Sudan Ebolavirus, mpox, and the Ukraine crisis. Beginning with a simple Excel spreadsheet for coding Ebola-related community feedback, we continued to add new features as needs arose, such as preloaded epidemic and health emergency coding schemes, preprogrammed results tables, step-by-step thematic analysis instruction, YouTube training videos, and planning and communication tools for effective use of the results.

**Implementation:**

The tool is a customized Excel workbook for qualitative text coding and thematic analysis that enables the user to code and derive key themes from texts, such as interview and focus group transcripts, notes, surveys with open-ended questions, and social media comments. We review the 10 programmed worksheets for planning, cataloguing, coding, and thematically analyzing any kind of text data.

**Conclusion:**

The strategies for rapid community feedback analysis during health emergencies are a special application of qualitative analysis methodology to the health emergency setting, enabling a deep reading and transparent and defensible interpretation of the text. Skills learned while using the tool are easily transferable to analyses using licensed software or fully manual methods. The tool offers a step-by-step guide for anyone to analyze text data to answer a relevant question in or outside the context of emergencies.

## Abstract (precis)

The CDC Excel Tool for Thematic Analysis is a free qualitative analysis tool designed for analyzing community feedback rapidly during epidemics and health emergencies that has proven effective in quickly generating actionable insights for decision-making.

## Key Messages

- The free U.S. Centers for Disease Control and Prevention Excel Tool for Thematic Analysis fills an important gap within global emergency response by offering self-paced user trainings, epidemic-specific coding schemes, and several other features not typically provided with other qualitative analysis applications.
- We detail 6 instances in which users provided actionable recommendations for outbreaks of Ebola, mpox, and dengue.
- An unexpected mixed methods use for the tool has emerged through the development process for coding responses of open-ended questions in surveys. With more advanced features of the tool, the user can both thematically analyze open-ended survey responses and link them to individual respondents for quantitative analysis.
- The tool makes it easier for ministries of health and global partners to incorporate community views into epidemic response and systematically follow changes in public sentiments over time.

## INTRODUCTION

It is well-documented that community attitudes and beliefs drive behavior.^1^^,^^2^ A recent metanalysis evaluating the empirical evidence behind COVID-19 pandemic-related policy recommendations demonstrated the influence of public sentiment on behavior during public health emergencies.^3^ Ruggeri et al. reported^3^:

*Claims suggesting trusted leaders and positive social norms increased adherence to behavioral interventions also had strong empirical support, as did appealing to social consensus or bipartisan agreement.*

Similarly, reports from several media types cite instances of COVID-19 (and other health emergency) control efforts that were less effective due to poor public participation resulting from a mismatch between approach and community needs or preferences.^4^^–^^9^

The challenge for public health authorities is to quickly understand the most influential perceptions, needs, and concerns during a health emergency and how community interventions can be structured to be successful within a given social and cultural environment.^10^ One way that epidemic response professionals can better anticipate public perceptions and beliefs and anticipate important constraints for intervention implementation is to begin with listening, that is, by collecting information from the public (through focus groups, unstructured interviews, public “town hall” listening sessions, and even public opinion surveys) about how they view the outbreak or emergency, what their primary concerns are, and what opportunities for action show the best promise for robust public participation. In these kinds of qualitative data collection strategies, the data consist of text, typically in the form of transcripts, meeting notes, online posts, open-ended survey responses, and notes in administrative records. Some strategies include facilitated listening, with questions posed, while others are unguided.^11^^–^^13^ Listening can also be done after the fact, by reviewing news stories or social media posts, records of public comments, complaint boxes, or phone comment lines—anywhere that people have been given an opportunity to freely express their concerns or points of view. In some cases, even survey questions can be considered community listening, if they are both open ended (answer in your own words) and broad enough to not prevent the participant from talking about what they think or feel is most important (e.g., What are your comments, concerns? How are you feeling about [x], and why?).

One factor that has hindered rapid qualitative assessment is the lack of clearly defined methods and tools appropriate to an emergency that can be implemented quickly and do not require a high degree of prior training to use. The U.S. Centers for Disease Control and Prevention (CDC) Excel Tool for Thematic Analysis is a newly published Excel workbook for qualitative text coding and thematic analysis, available in English and French. The tool is designed to help users simply and quickly analyze texts to understand public concerns, questions, and perspectives during an outbreak or other public health emergency and has predefined text-coding schemes for these scenarios. However, it also provides space for users to furnish their own coding scheme and can be easily adapted for analysis in domains outside of emergency responses and even public health. Commercially available qualitative analysis software provides a platform for coding and thematic analysis but does not lead the user step-by-step through how to code and create themes. A unique feature of the CDC tool is that it teaches the concepts of text coding and thematic analysis as users enter and organize their text. No prior experience with thematic analysis is required. This tool is free and is designed for novice analysts, with tutorial YouTube videos.^14^

### Theoretical Underpinnings of the Tool

The use of qualitative analysis in public health has a long history^15^^–^^17^ and is favored for describing phenomena in a nonnumerical way,^18^^,^^19^ often to understand context, relationships between individuals, and points of view that have yet to be discovered.^20^ Within qualitative analysis, there are numerous theories and approaches. While thematic analysis has been associated with a number of different theories and approaches^21^^–^^23^ (and the tool may be used in different ways), for its most typical use (rapid analysis of community perceptions and concerns), the tool uses the qualitative descriptive approach.^24^^–^^26^ This approach is known for describing, rather than explaining, phenomena^24^ and has at its core the amplification of the voice of the community rather than creating or confirming theories of why they hold the views they do. As Hall and colleagues described^27^:

*Distinct from its counterparts, [qualitative description] is not focused on deep description as in ethnography, theorization like grounded theory, or recontextualization and composition typical of other qualitative methods. Instead, [qualitative description] is rooted in the direct and rich description of experiences or events, maintaining a close proximity to the data without straying into extensive theorization or abstraction.*

Qualitative description also acknowledges the subjectivity inherent in qualitative data interpretation. Researchers strengthen its quality by following a structured, logical, and transparent approach and ensuring that the plan includes informed consent, as appropriate, and plans for sharing analysis results with the participants.^28^

We describe a tool that merges a structured, theory-based approach with ease of use so that analysis can be quickly performed to aid in response. The strategies contained in the tool are straightforward and could be used with a different software product or even with pen and paper. We describe the tool, review the history of its development through multiple epidemic responses that drove the addition of new features, and provide examples of how it has been used in emergency response and other settings.

## TOOL DEVELOPMENT

The tool was developed over the course of 6 emergencies within 5 years, as social and behavioral scientists at CDC responded to requests from epidemic response professionals for a way to quickly integrate community input into emergency response decision-making. The 6 emergencies included:

- Ebola response, Democratic Republic of the Congo (DRC) (2018–2020)
- Ebola response, Guinea and DRC (2021)
- COVID-19 pandemic response (2020–2021)
- mpox response, United States (2022)
- Sudan Ebola response, Uganda (2022–2023)
- Ukraine regional response and recovery (2022)

Requests varied based on the situation, which led scientists to revise and expand the tool’s capabilities to respond to new needs and challenges as they emerged (Table). The tool began as a simple Excel spreadsheet for coding Ebola-specific community comments and grew into a multipurpose planning, coding, and thematic analysis tool.

**TABLE.** Phases of Tool Development Corresponding to Health Emergency Responses, 2018–2023

	**2018–2020**	**2020–2021**	**2022–2023**	**2023**
Health emergency and location	Ebola outbreak in northeastern DRC	COVID-19 in Africa and Asia	Ebola outbreaks in northeastern and western DRC; Sudan Ebola virus in Uganda; mpox in the U.S.	Crisis management support training for health officials from Ukraine, Poland, and other neighboring countries
Tool development steps	Created initial coding tool to assign codes to thousands of comments from Ebola-affected communities	Restructured initial coding tool to fit more outbreak contexts and simplify analysis by topic	Added thematic analysis component to the tool, with step-by-step instructions	Streamlined and enhanced the user experience; Added health emergency coding scheme, ability for user to create own coding scheme, and planning worksheets
Need that it met	Red Cross volunteers needed a system for quickly categorizing thousands of public comments in Ebola-affected communities	International partners and CDC colleagues needed a tool for quickly categorizing text from a range of outbreaks	Field users needed to conduct their own analysis rapidly and independently	Training workshops revealed a need for thematic analysis for broader types of health emergencies, improvements to the user interface, and planning tools to help users get started

Abbreviations: CDC, Centers for Disease Control and Prevention; DRC, Democratic Republic of the Congo.

### Creation of Initial Coding Tool, 2018–2020

In August 2018, the first cases of Ebola virus disease were confirmed in the northeastern region of DRC.^29^ By April 2019, cases had climbed to more than 100 per week,^30^ frustrating efforts to control the outbreak. Government and international response workers were regarded with fear and suspicion^31^ while widespread political violence limited travel and tracing of contacts.^32^ The International Federation of the Red Cross (IFRC) and the Red Cross Society of DRC (Red Cross) deployed staff and local volunteers to make door-to-door visits in communities with cases. As part of its commitment to public accountability,^33^ the Red Cross recorded any questions, comments, suggestions, or complaints from the public during their encounters.^31^

Although the Red Cross (with support from IFRC) had a well-established process for building rapport and recording comments and questions from community members, there was no structure in place for rapidly aggregating the comments to identify patterns and trends. We assisted by coding a series of “drop-down” menus to hold sets of codes that corresponded to the sections of the Red Cross notes sheets (“questions,” “statements,” “suggestions,” and “appreciation”). Using basic Excel functions such as pivot tables and chart generators, the team created more than 80 reports of frequent and notable comments (1,000–3,000 comments per report) over a period of 18 months that were each returned to Red Cross within roughly 2 weeks of data collection. They were then able to review this feedback as they planned future community information sessions.^31^ After training and CDC capacity building support, by the following year DRC staff were using the system entirely on their own, and IFRC further developed the Excel spreadsheet into a dashboard. As a final step, CDC, IFRC, and the Red Cross conducted a final comprehensive analysis of the more than 350,000 comments collected between 2018 and 2020 to identify trends and patterns in public attitudes.^31^

### Creation of a Universal Epidemic Coding Scheme, 2020–2021

As part of its COVID-19 support to Red Cross societies worldwide, IFRC assisted in analyzing community feedback data. CDC provided a community feedback coding scheme, similar to what had been developed during Ebola but specific to COVID-19. This was used to code more than 110,000 individual community feedback comments from 25 countries.^34^ In providing this adaptation, we noted that much of the coding scheme remained the same despite the difference of pathogen. For example, both coding schemes were framed around 6 main areas of community comments during an epidemic:

1. Feedback relating to the nature or characteristics of the disease
2. Details about the location and the progression of the outbreak
3. Reactions or comments about others’ reactions to the outbreak
4. Community reactions and observations about the government-led response
5. How people are experiencing illness and recovery
6. Feedback about other issues not directly related to the first 5 categories

After providing this adaptation to IFRC, we determined that it would be strategic to create a “universal” epidemic community feedback coding scheme that could be used for any kind of infectious disease outbreak. This coding scheme was housed in an Excel file, with instructions for importing text into rows in the worksheet and assigning codes to segments of text. We tested and refined it by coding text related to different infectious diseases (Ebola, COVID-19, Zika, Lyme disease), and from different types of community listening (in-person comments, focus groups, in-depth interviews, notes from community meetings). The new coding scheme was conceived somewhat differently than the original design (that followed the Red Cross data collection form), and it also included a way to “flag” comments for immediate follow-up (for example, reports of unsafe situations, critical misinformation, or implementation problems with response efforts) independent of the coding scheme. This “flagging” function was included because past emergency responses had demonstrated that community feedback can be the earliest signal that response efforts in communities are not accomplishing their goals. For example, during the Zika response in Puerto Rico in 2016–2017, community feedback mechanisms identified that Zika testing for pregnant women and distribution of “prevention kits” (including insect repellent, larvicide for standing water, and other materials) were not functioning properly.^35^ This information instigated actions to correct problems immediately. With these kinds of situations in mind, having a system for flagging comments that should be shared with program officials immediately and independently from the main analysis can be tremendously helpful.

### Addition of Thematic Analysis Process, 2022–2023

In 2022, we were asked to provide qualitative analysis for a series of 36 key informant interviews with meat processing workers and community leaders in a community surrounding the facility that was experiencing many COVID-19 cases. The project was led by field epidemiologists who were not experienced in analyzing these kinds of interviews. We used qualitative analysis software for the text coding and mostly manual processes for development of themes. When this was complete and the results shared, several more epidemiology field teams requested the same thematic analysis assistance for similar community interview projects but given the small size of our team, it was not possible to meet the demand. However, we were able to provide a brief training in coding and thematic analysis to the requesting teams so they could undertake the work themselves. Since these staff did not have access or abilities to use qualitative software but did have access to and experience using Excel, we clearly saw the need for a simple tool for both coding and thematic analysis using Excel that was customized for use in epidemic response.

Building on our experience teaching thematic analysis, we designed a set of worksheets in Excel that walk the user through viewing pivot tables made from text codes (and quotes from the text) to drafting and supporting representative themes. We gathered feedback on ease of use, the clarity of the instructions, and on different ways of teaching people how to use the tool (e.g., written in the worksheets, separate instruction guide, YouTube training videos).

### Addition of Planning Worksheets and Improved Ease of Use and Visual Appeal, 2023

In June 2023, with support from the CDC Ukraine response, we co-led a week-long training for health officials from 11 nations in risk communication and community engagement strategies for emergencies (Krakow, Poland, June 2023)^36^ and introduced the tool. In preparation for the training that included simulated exercises, we created an “other health emergency” text coding scheme to capture feedback during emergencies in which health threats were a component of the emergency but not the entire emergency. In addition, a completely blank coding worksheet was added, so that users could make a fully unique coding scheme.

Once the tool contained 3 coding schemes and several separate supporting worksheets for each coding scheme, the visual layout of the tool became complex. We inserted “coding scheme selection” buttons (using Excel VBA programmed macros) so that the number of visible worksheets would be cut down to 10 based on which coding scheme the user chose. The overall look of the worksheets was revised by moving much of the written instructions to YouTube tutorial videos.^14^

Observation of the simulated emergency exercises during the training revealed that participants struggled with getting started with the community listening process, which requires being able to clearly articulate why you are doing the listening, what the community perceptions, behaviors, or concerns are that you are trying to understand, and how this knowledge will aid in ending the emergency. The inquiry planning worksheet includes several questions that help the analyst think through what they hope to gain from undertaking the collection and analysis and how the results might be applied to an outbreak or other emergency. This planning step is critically important for assuring that the time spent produces useful information that contributes to ending the emergency. Similarly, the action planning worksheet prompts the user to plan how the findings will be used in the response.

## IMPLEMENTATION

**FIGURE 1:**
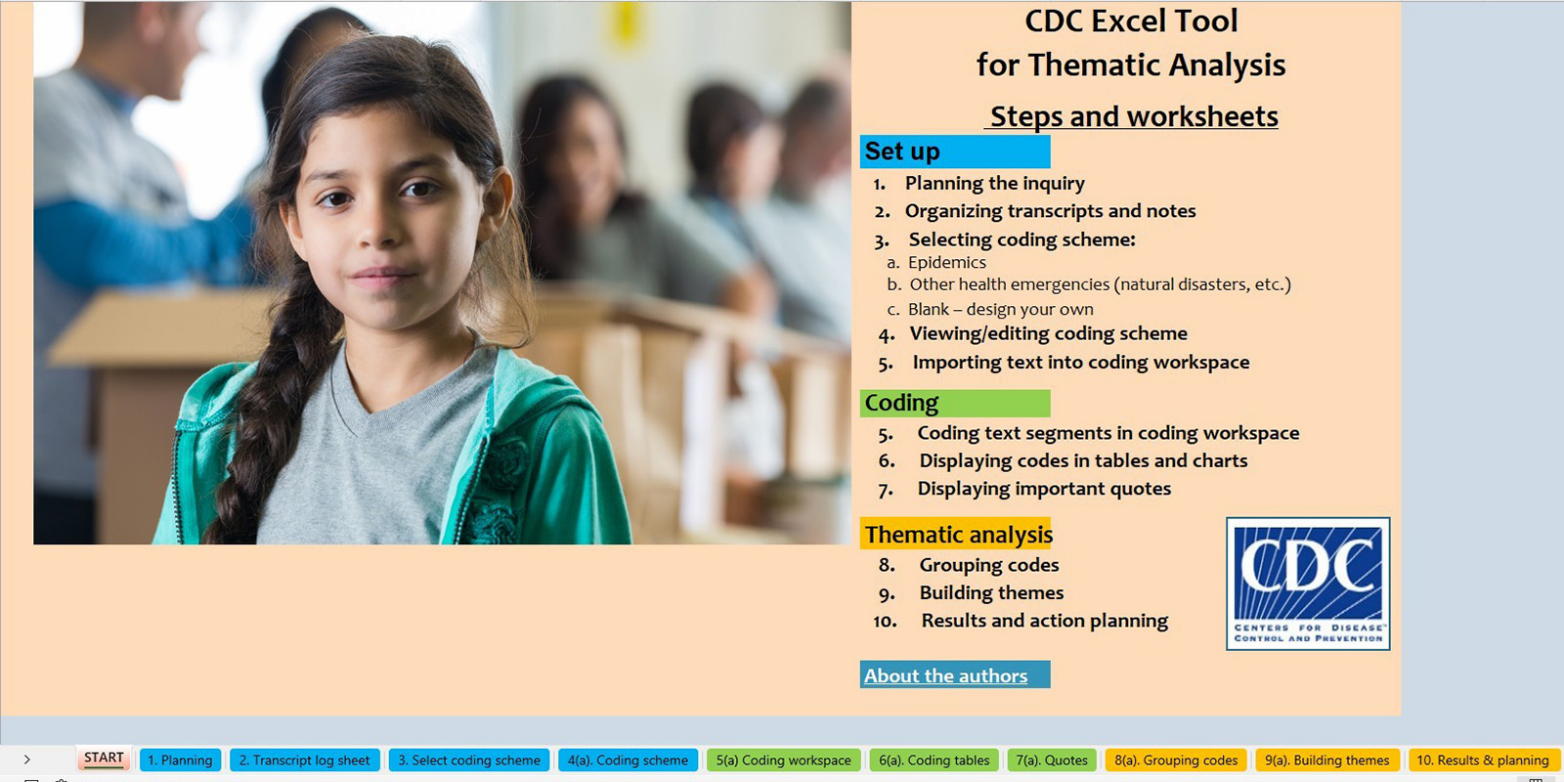
Start Page for the CDC Excel Tool for Thematic Analysis The tool contains 10 worksheets grouped into 3 sections of Set up, Coding, and Thematic analysis.

The tool consists of a “start” overview worksheet (Figure 1) and 10 color-coded planning and analysis worksheets grouped into set up, text coding, and thematic analysis sections or phases.

### Set-Up Phase

The first step in using the tool is to define the purpose of the inquiry on the “1. Planning” worksheet. For example, the analyst may wish to understand the reasons for low participation levels in public infection control efforts or to assess commonly used information sources during a health emergency. A clearly stated purpose helps the analyst identify relevant themes later and increases the likelihood that the inquiry can inform useful action toward reducing disease transmission or improving actions of responders. This step can be undertaken before collecting data or when preparing to review existing texts.

Once the user has collected one or more transcripts or texts of some other kind (e.g., notes, social media posts), the “2. Transcript log sheet” worksheet can be used for cataloguing them and recording key attributes (e.g., date collected, number of participants, demographics). This provides an annotated table of contents of the texts included in the analysis. The texts initially will be in separate word processing documents but later input into the coding workspace where they will remain. This is particularly useful when analyzing several different listening texts at the same time, for example, multiple interviews, listening sessions, social media posts, and meeting notes.

In the set-up phase, the analyst also chooses which text coding scheme they will use by opening the “3. Select coding scheme” worksheet and selecting one of 3 buttons: epidemic, other health emergency, or blank coding scheme. While users are encouraged to customize their coding scheme as appropriate, for users working in epidemic response or with another kind of health emergency, having a coding scheme to start with can save time. With the third blank coding scheme, users build their own scheme. In the field of qualitative analysis, text coding schemes are either developed before analysis, based on analytical questions (deductive coding), or as the analyst begins coding (inductive coding).^37^^,^^38^ The tool allows for both approaches.

Once the user has selected the preferred coding scheme, a set of new worksheets appears, starting with “4. Coding scheme, which contains a list of all the codes corresponding to the selected coding scheme. The user can select a different coding scheme at any time, which will result in the “4. Coding scheme” worksheet changing.

The final step in the planning phase is for the user to paste their transcripts or other texts into the “5. Coding workspace,” arranging the text so that every sentence is in its own row. This is necessary because Excel does not allow the user to highlight part of the text to assign a code. The entire contents of a given cell must be coded. Therefore, splitting the text into rows so that there is one sentence per row allows for coding text strings with the least amount of splitting or merging text. Once this has been completed according to the directions, the user is ready to begin coding.

### Text Coding Phase

This phase involves assigning codes (short, descriptive labels) to segments of the text according to their meaning and how they relate to the purpose of the inquiry. This type of coding is known as “descriptive,”^39^ because the goal is to code according to the meaning of the text segment. In qualitative analysis in general, there are numerous other ways to code data (e.g., emotion coding, values coding, versus coding).^39^ However, for the purpose of quickly summarizing community comments during an emergency, descriptive coding has proven to be most useful. Experience has also shown the utility of a two-level coding system: (1) a “broad code” indicates the general topic being spoken about, and (2) a more “specific code” provides a more detailed classification.

**FIGURE 2:**
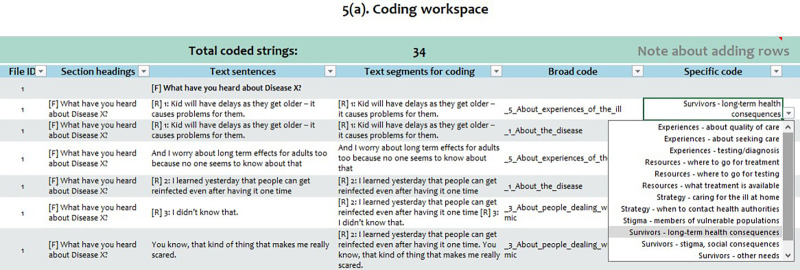
Coding Assignment Worksheet in the CDC Excel Tool for Thematic Analysis Users assign codes to the text using easy-to-use drop-down menus.

To code a segment of text, the analyst reviews the text sentence in each row and first decides whether the sentence needs to be broken apart so that subsections of the sentence can be coded in different ways. Alternatively, the analyst can decide to merge 2 or more sentences and assign a single code to all merged sentences. These adjustments are made in a column to the right of the original text. When the text is organized into segments appropriate for coding, the user enters a broad code from a drop-down menu in the next cell to the right and then selects from a second drop-down menu in the next cell to the right on that same row, selecting a specific code to describe the text (Figure 2).

This two-tiered descriptive coding system makes it easy to move back and forth between broad groups of text and more specific groupings. The user also has the option of duplicating a sentence or part of a sentence to assign multiple codes to it.

When the assignment of the broad and specific codes is complete, the user has the option of assigning a third code. This coding uses a separate coding scheme, which has been customized by the user to “flag” the text segment for immediate follow-up, whether due to misinformation, indications of possible intervention implementation failures, or other issues determined by the user to warrant immediate action. When the user has either coded this way or decided that the flag code is not needed, they repeat these steps for the next row until all the text has been reviewed and assigned codes appropriately. For more advanced users, any of the 3 coding schemes can be easily copied from the coding scheme worksheet and used with other qualitative analysis software. The epidemic coding scheme has been tested numerous times with a range of outbreaks and thus will provide a good model for users of any software.

**FIGURE 3:**
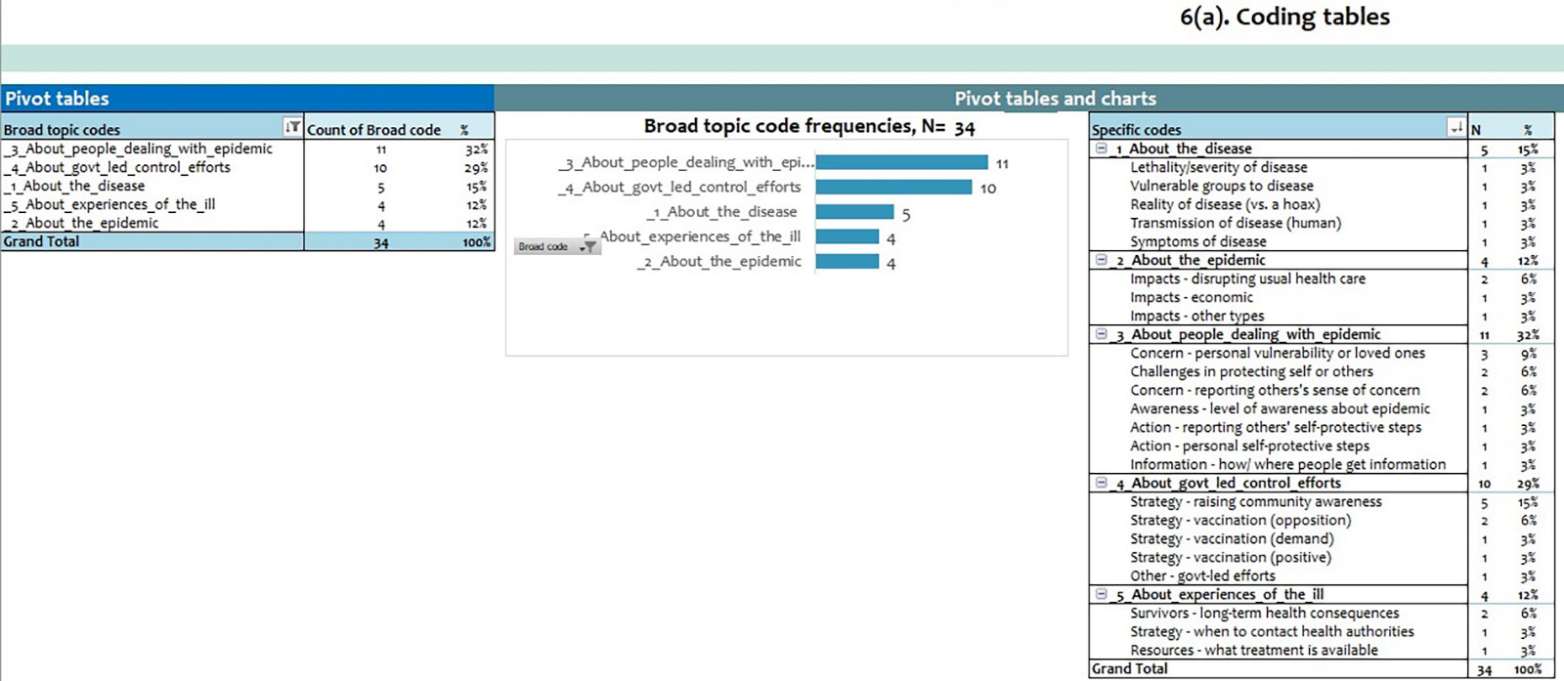
Coding Summary Worksheet in the CDC Excel Tool for Thematic Analysis The tool includes a summary sheet that displays frequencies of the user-assigned codes.

Once all the relevant text has been coded, the worksheet, “6. Coding tables” will display a numeric summary of the codes in a table and chart, with the most frequently assigned codes at the top (Figure 3). Similarly, the “7. Quotes” worksheet displays the text segments grouped by their codes. This worksheet allows the user to filter by code to limit the number of codes viewed at one time. This feature is particularly important for sharing verbatim text segments to exemplify codes and themes.

### Thematic Analysis Phase

The thematic analysis phase walks the user through creating themes using 2 worksheets, “8. Grouping codes” and “9. Building themes.”

**FIGURE 4:**
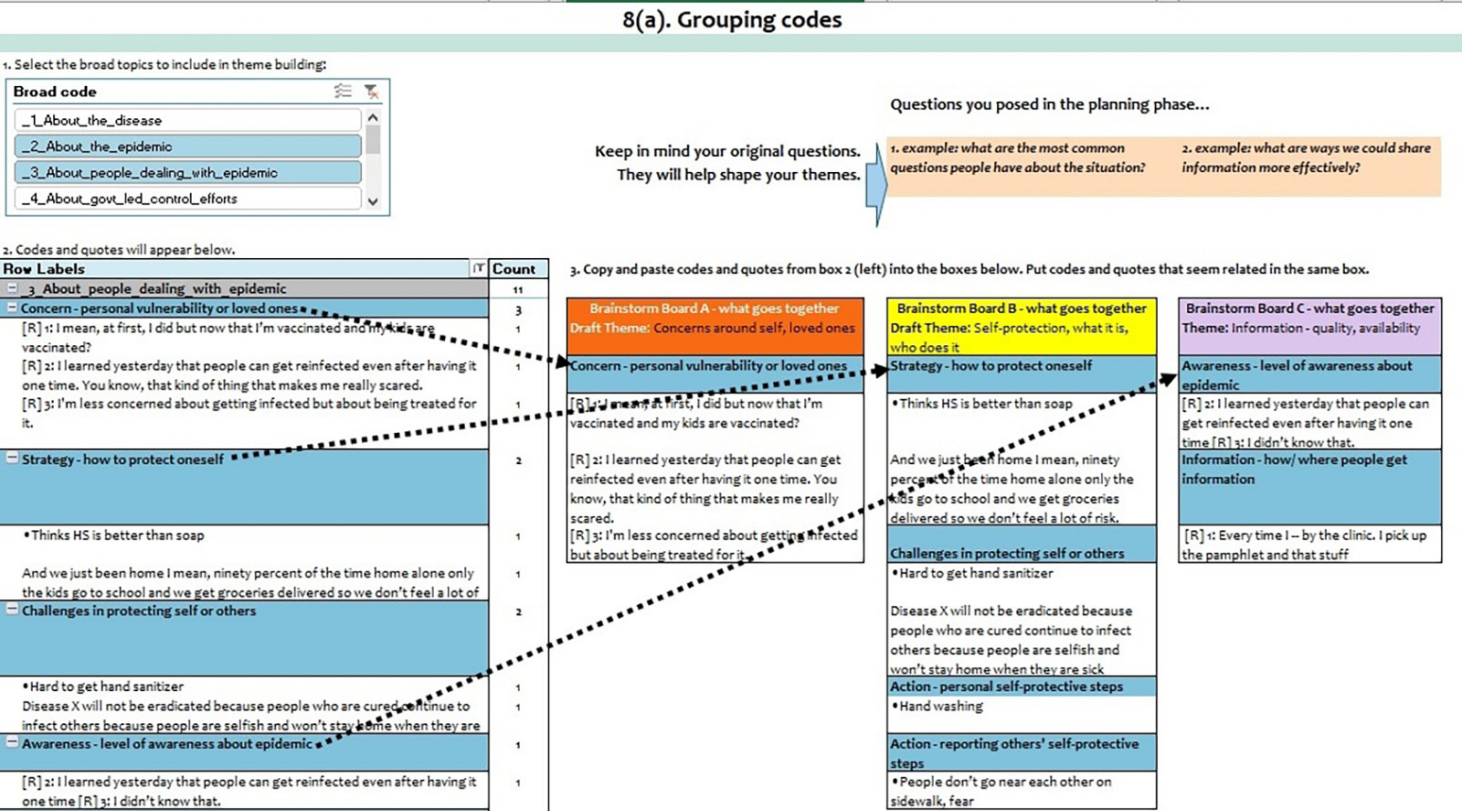
Brainstorming Boards Worksheet in the CDC Excel Tool for Thematic Analysis In the tool’s final thematic analysis phase, users can group the codes with the associated quotes and start building themes.

The “8. Grouping codes” worksheet displays a table of the most common codes and the accompanying text segments. This table also has filters for limiting the number of codes being reviewed at any one time, so the user can focus on a particular area of interest and review other sets of codes separately, which can be particularly useful for large text datasets. Figure 4 shows the “8. Grouping codes” worksheet with example text data. This is the first of 2 thematic analysis worksheets.

In Figure 4, only one broad code has been selected in the filter box (or “slicer”) for theme building: “3. About people dealing with the epidemic.” Consequently, the table just below it displays only the specific codes (highlighted in blue), that were used under broad code 3. Below each specific code in blue are all the text segments that have been assigned that code.

To the right of the pivot table, there are 3 columns (A, B, and C) that serve as “brainstorming boards” (users can add more columns). The arrows in the figure show where codes (with their text segments attached) have been placed on the boards. In Figure 4, the example shows that the user has come up with 3 initial ideas for themes: concerns around self, loved ones; self-protection-what it is and who does it; and information quality and availability. When all the codes of interest are placed on a board, the worksheet is done.

**FIGURE 5:**
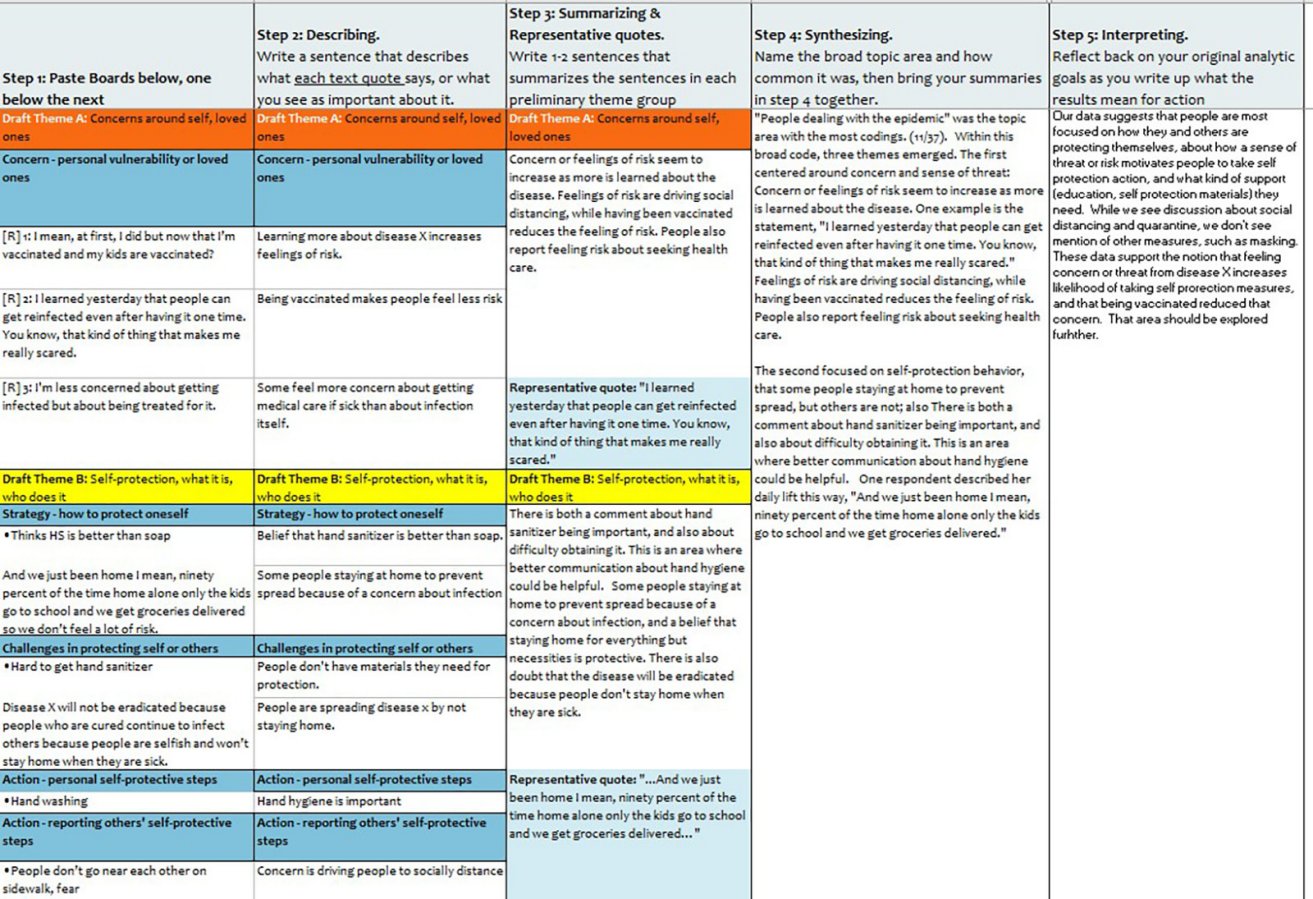
Themes Worksheet in the CDC Excel Tool for Thematic Analysis The final worksheet of the tool walks the user step-by-step to summarize the final themes, select representative quotes, and interpret the results in relation to the initial analytic inquiry.

The “9. Building themes” worksheet uses the boards created on the previous worksheet to help the user work through the remaining steps in building final codes and interpreting the results. In the illustrative example in Figure 5, the user has copied and pasted boards A, B, and C from the “8. Grouping codes” worksheet, vertically in column B. Aligned this way, the user can proceed across the table, following the instructions in each column heading. These steps assist the user in processing the groups of coded text and interpreting their meaning to synthesize it with a brief narrative description and sample quote.

Once the user has completed all the steps shown in the table and has created a narrative summary of the themes, the last step is to re-read the initial inquiry questions, which are reprinted in this worksheet (not shown) and to align the original questions and the answers that your interpreted results offer to them. In this example, the original inquiry questions were about what needs for information and communication did the public have surrounding an imaginary “disease X.” From the text analysis, we can see that there was inconsistency in social distancing and a perception that hand sanitizer was important but not available. These findings would be quite relevant to questions about information needs.

The final worksheet, “10. Results and planning,” is formatted much like worksheet 1, with a form to complete, only this time including the brief answers to the original questions, along with planning questions about what will be done with the results.

## USE CASES OF THE CURRENT TOOL

In 2022, the tool began to be used in international epidemic responses. For example, during the 2022 Sudan Ebola outbreak in Uganda, Zalwango and colleagues interviewed Ebola survivors, their families, district health officials, and community health workers.^40^ Using the tool methodology, they found that in addition to stigma associated with fear of infection, Sudan Ebola survivors also experienced stigma resulting from well-intentioned assistance efforts, such as “home visits by health workers, public distribution of support items, and conspicuous transport from home to the survivor’s clinic …” This finding has important implications for future Ebola survivor support efforts.

Another example is the use of the tool for quickly identifying common community questions and concerns as part of a CDC-led, hybrid (in-person and Zoom) public meeting for Ugandans traveling to the United States and their families. Due to concerns at the time about importation of Sudan Ebola, U.S. officials were routing all travel from Uganda through a limited number of airports, which then screened arriving passengers for Ebola symptoms. Findings from this thematic analysis included signals of mistrust of airport screenings among some, with offers to act as liaisons to the Ugandan community to build collaboration from others (Supplement 1).

Earlier in 2022, CDC used the tool as part of the mpox clade II response in the United States in 2 different ways. First, as mpox cases were first being identified, predominantly among men who have sex with men, there was concern that news coverage might be inaccurate and stigmatizing to those most at risk. A thematic analysis study of news media coverage in New York State, one of the highest incidence regions of the country, showed that while the coverage was accurate overall, the emphasis on transmission among this one group, coupled with little to no information about self-protection may have unintentionally contributed to stigma (Supplement 2). The tool was also used as part of a mixed-methods survey analysis of the public regarding mpox awareness and disease knowledge. In this case, the tool was used to analyze survey responses written in respondents’ own words rather than by selecting from a response list. This kind of response is extremely useful for open-ended questions because responses are not influenced with a list of response options. Consistent with the findings from the news media analysis, respondents reported having heard about groups at highest risk most frequently (20% of respondents), with the increasing number of cases and mpox’s infectiousness being the next most frequent responses (Supplement 3).

In 2024, the tool was posted on the CDC website and made freely available for download, with several YouTube training videos provided. In the current clade I mpox response in DRC, the tool has been used to rapidly analyze community feedback from affected communities to inform vaccination promotion approaches (personal communication, Henriette Bulambo, November 23, 2024). Responders to the dengue-oropouche outbreak in the Caribbean have also recently used the tool to rapidly analyze in-depth interviews with clinicians to understand their needs to appropriately respond to the increase in cases (personal communication, Valerie Mac, November 20, 2024).

## DISCUSSION

Our experience serving as social scientists for multiple health emergencies around the world enabled us to develop several distinctive features of the CDC Excel Tool for Thematic Analysis: it uses a free, widely available platform (Excel); a universal “starter” coding scheme for epidemic-related text coding; preprogrammed coding and display tables; easy-to-use thematic analysis tools; and planning and reporting tools that center the analysis on questions that really matter during an emergency.

In the early days of developing the tool, we encountered a pervasive belief among epidemic response leaders that studying community perceptions and adapting strategies to better fit community desires was too time consuming and could not be effectively integrated into epidemic response strategies. However, the experience of the 2018–2020 Ebola response in DRC demonstrated that opposition to response efforts only decreased after investments were made in listening and responding to what was learned.^31^ It appeared that not investing in community listening perpetuated the outbreak by not addressing community opposition to control efforts.^41^

CDC, IFRC, and WHO are not alone in recognizing the larger role that community feedback and community engagement should play in epidemic and health emergency response. Across the health emergency and humanitarian space, there is now a greater recognition of the importance of meaningfully and quickly incorporating community perspectives into emergency response efforts through rapid community listening and social science analytics.^31^^,^^41^^–^^45^ The field of social media text data analysis has grown tremendously, as evidenced by the World Health Organization’s roll out of the concept of an infodemic, defined as “…too much information including false or misleading information in digital and physical environments during a disease outbreak”.^46^ The IFRC has developed a community feedback toolkit,^47^ containing 34 print tools for running a community feedback and accountability program. While the importance of effective listening to community perspectives is emphasized in several reviews,^48^^,^^49^ we did not find any free tools that combined a coding and thematic analysis methodology with software for performing the analysis.

### Limitations of the Tool

One of the main strengths of the tool, that it is in Excel (making it free and in a format that many people are already familiar with), also presents some limitations. Software that is specifically created for thematic analysis typically allows the user to code text segments in the text without having to split up the text into separate cells. For users whose text data is not divided into rows already (e.g., social media posts and short comments), this requires some formatting not required in licensed software such as MaxQDA or NVivo.

In addition, the tool was created in an end-user version of Excel, and thus there is always the possibility of damage to the programming or structure of the file by users. To make it fully customizable, few tool features are locked. Currently, we recommend that users start new projects by first saving a copy of the original downloadable file and if they encounter errors, to restart their work in a new version of the file. A related limitation is that thematically analyzing extremely large datasets can be challenging, due to the sheer number of text segments. This problem can be mitigated by thematically analyzing a limited number of coding groups (or a single broad code) at a time, and then combining them at the end, or by randomly pre-selecting a limited number of text segments within each code to use for theme building. A system that could reveal and hide different sections more easily would facilitate theme building. Similarly, there is not yet an automated function for comparing coding between analysts. It is possible to insert additional coding columns to the “*5*. Coding workspace” worksheet so that a second coder can duplicate the coding (with the original coding hidden); however, the original coder will have specified the text segments (e.g., single sentences, partial sentences, multiple sentences), and will have duplicated some segments to code them multiple times before they begin coding. Thus, when multiple coders are working on the same data, one coder might first establish the segments to be coded, and then multiple coders could separately code those segments. This is not as simple as some licensed software (e.g., Dedoose). Alternatively, 2 coders could work entirely separately, and compare themes at the end of their process. In the future, a more automated process for comparing set up and coding between multiple analysts would be helpful.

Lastly, it remains unknown what data capacity the tool can accommodate, and whether embedded macros will have challenges over time. The largest dataset analyzed to date had more than 100,000 rows; however, the durability and stability of the tool are as yet unexplored.

## NEXT STEPS

The tool is currently being studied using past data to determine how artificial intelligence can be incorporated to speed up coding processes and how it can augment theme building by detecting patterns in coded data (or uncoded text) and applying those patterns to answer the user-defined analytic questions. The goal is to augment, not replace, manual analysis processes^50^ and offer multiple ways of viewing the data from which the analyst could choose the perspective most appropriate to the context. While a French-language version is available upon request, next steps include providing French- and Spanish-language versions online for free download, and to develop further training tools.

## CONCLUSION

The CDC Excel Tool for Thematic Analysis assists with authentic community engagement during health emergencies by providing a tool for ministries of health and global partners to efficiently and effectively analyze qualitative data in a rigorous and transparent way. For partners focusing on capacity building, the tool also facilitates collaboration with partners who may not have the resources to invest in licensed software applications. Ultimately, our aim is to make this type of analysis (and epidemic-specific coding schemes) accessible to a wider range of public health professionals, other analysts, and the public. In the long run, this will assist in helping public health action incorporate public needs and viewpoints more effectively.

## Notes

**Peer Reviewed**

**First published online:** November 4, 2025
